# Two logics of policy intervention in immigrant integration: an institutionalist framework based on capabilities and aspirations

**DOI:** 10.1186/s40878-017-0064-0

**Published:** 2017-12-07

**Authors:** Philipp Lutz

**Affiliations:** University of Bern, Fabrikstrasse 8, 3012 Bern, Switzerland

**Keywords:** Immigrant integration, Assimilation, Multiculturalism, Capabilities, Aspirations

## Abstract

The effectiveness of immigrant integration policies has gained considerable attention across Western democracies dealing with ethnically and culturally diverse societies. However, the findings on what type of policy produces more favourable integration outcomes remain inconclusive. The conflation of normative and analytical assumptions on integration is a major challenge for causal analysis of integration policies. This article applies actor-centered institutionalism as a new framework for the analysis of immigrant integration outcomes in order to separate two different mechanisms of policy intervention. Conceptualising integration outcomes as a function of capabilities and aspirations allows separating assumptions on the policy intervention in assimilation and multiculturalism as the two main types of policy approaches. The article illustrates that assimilation is an incentive-based policy and primarily designed to increase immigrants’ aspirations, whereas multiculturalism is an opportunity-based policy and primarily designed to increase immigrants’ capabilities. Conceptualising causal mechanisms of policy intervention clarifies the link between normative concepts of immigrant integration and analytical concepts of policy effectiveness.

## Introduction

Immigration is a force of social change and has contributed to reinforce ethnic and cultural diversity of many Western societies. Over the last three decades, the integration of immigrants has become a salient political issue in most European countries and policy makers implemented targeted policies to foster integration of new immigrants (Givens, 2007). Integration policies are meant to strengthen social cohesion and the performance of the immigrant population. However, design and effectiveness of these policies vary greatly between countries (see Brubaker, 1992; Joppke, 1999; Favell, 2001). What is more, scholars as well as policymakers disagree on how integration policies should be designed in order to achieve favourable policy outcomes. Large parts of the fierce debates about the merits and shortcomings of integration policies have been occupied with assimilation and multiculturalism as two competing understanding of the integration process (Givens, 2007; Koopmans, 2013; Vertovec & Wessendorf, 2009). While assimilation stresses the importance of immigrants to adapt to the mainstream society, multiculturalism stresses participation of immigrants with the preservance of cultural distinctiveness. These two broad approaches to the integration of immigrants are not only concepts about the nature of the integration process but also specific types of policies (Koopmans, Statham, Giugni, & Passy, 2005). While policies of multiculturalism have been found by some scholars to yield favorable integration outcomes (e.g. Bloemraad, 2006), others considered the same policies to have been a responsible factor for integration failures, and evaluated policies based on immigrants’ assimilation as more effective (e.g. Koopmans, 2010). Despite the surge in comparative research on immigrant integration policies, the notions of integration as well as different understandings of it such as assimilation and multiculturalism remain highly contentious within academia. Furthermore, the bulk of empirical studies resulted in contradicting empirical findings (see Goodman, 2015; Helbling & Michalowski, 2017, for an overview). While there is a vast and growing literature theorizing on normative and descriptive types of integration policy regimes, the question of what mechanisms of policy intervention are implied by these policies is rarely addressed in comparative studies. The process of policy intervention remains often vague and underspecified (Castles, De Haas, & Miller, 2014). The lack of a coherent framework of causal mechanisms behind the integration of immigrants prompted scholars to call for an increased analytical rigor in order to better understand the process of policy intervention (Goodman, 2015). To delve into this blackbox this article extends the existing typology of assimilation and multiculturalism with their assumed causal mechanisms. This step allows translating normative policy regimes into an analytical framework that is susceptible of testing policy effectiveness in a more systematic manner. For that purpose, immigrant integration outcomes are conceptualised as a function of both integration *capabilities* of immigrants, and their integration *aspirations*.

On the methodological side, this article uses ‘actor-centered institutionalism’ as its framework of analysis (Scharpf, 1997) as integration policy outcomes are considered a product of policies as an institutional framework and immigrants as intentional actors. By integrating capabilities and aspirations into an institutionalist framework for integration policy outcomes, two distinct pathways of policy intervention are classified. Integration policies, when understood as institutions, constitute both an incentive structure and an opportunity structure that influences the capabilities and aspirations of immigrants. While institutional incentives influence the attractiveness of integration, institutional opportunities influence the feasibility of integration. This perspective is then applied to the literature on assimilation and multiculturalism to evaluate how their different institutional designs are assumed to influence the behaviour of immigrants. The results imply that these two regime types are based not only on different normative underpinnings and descriptive characteristics of policy outputs, but just as much on different recognition of the integration policy problem and distinct causal mechanisms of their policy intervention. The new framework substantiates the assertion that policies of multiculturalism recognise a lack of opportunities as the policy problem and the policy intervention is designed to increase capabilities, whereas policies of assimilation recognise a lack of motivation as the policy problem and the policy intervention is designed to increase aspirations. The capability-aspiration framework offers a heuristic to separate normative from analytical concepts, and facilitates a more rigorous testing of the effects integration policies have on outcomes. Clarifying causal mechanisms of policy intervention provides a fertile ground for the future study of integration policies.

This paper is structured in the following way. In a first step, the normative challenge in the current literature on immigrant integration is reviewed and the need for the conceptualization of causal mechanisms is discussed. Then I present a new institutionalist framework that proposes to evaluate the outcomes of integration policies as a function of integration capabilities and integration aspirations. In the next section follows an application of the framework to assimilation and multiculturalism as distinct types of integration policy regimes in order to derive two different logics of policy intervention. The paper concludes by assessing the benefits and implications of the proposed framework for future research.

## The normativity trap of integration policies and how to avoid it

Policies of immigrant integration share the aim of steering and guiding the integration process in a more favourable direction. Any attempt to study immigrant integration policies requires a particular conceptualization of integration including the normative assumptions that a particular policy is based on. As argued by Spencer and Charsley (2016), any understanding of the factors of influencing immigrant integration requires a concept about the nature of the integration process. In a similar vein, the enquiry of integration policy effectiveness requires a concept about the nature of policy intervention into the integration process. However, there is still a surprising lack in such conceptualizations in the existing literature. In the following literature review, I argue that the lack of linkage between normative concepts of integration and analytical concepts of integration policy intervention is an important pitfall in the comparative study of immigrant integration policies.

Although there is no universal definition of the term, the core meaning of integration is commonly described a social process of settlement and the accommodation by both the native and the immigrant populations, resulting in an increased social membership of immigrants (Givens, 2007, p. 72). The aim of integration is commonly described as achieving equal opportunities for immigrants within society (Brochmann, 1996; Entzinger, 1990; Kymlicka, 1995, pp. 15–17). However, the idea of integration often indicates what many citizens expect immigrants to do, and contains ideals of nationhood and society. Accordingly, the notion of immigrant integration is highly contested, and is often used as a normative idea instead of an empirical concept (see Bauböck, 2006).^1^ The best expressed are these normative expectations in assimilation and multiculturalism as two opposing modes of integration. Assimilation theories have been the forerunners in integration studies defining integration as a linear process in which immigrants were supposed to adapt themselves to the mainstream society (e.g. Warner & Srole, 1945). In this perspective, immigrants are perceived deficient and therefore supposed to learn the way of life in the receiving country. Assimilation is based on the general expectation that immigrants should adapt to the new culture and abandon their own traditions and habits in a way that the receiving society remains relatively unchanged by immigration. This ‘ethnocentric’ view faced harsh criticism for being one-sided, creating an illusion of a homogenous society and ignoring structural inequalities (Safi, 2011). More recent conceptions of assimilation define the concept in more empirical terms as the process of becoming more similar to the majority of society (e.g. Alba & Nee, 1997; Rumbaut, 1997). The conceptual core of assimilation theories remains the adaptation of immigrants with the aim that differences with the majority population disappear over time.

As an opposing mode of integration, the concept of multiculturalism has become an important focus of scholars and policymakers.^2^ Multiculturalism defines integration as the participation of immigrants on equal terms while preserving their cultural distinctiveness (e.g. Kymlicka, 1995, 2001). Theories of multiculturalism aim to accommodate diversity and ethnic minorities by creating ‘polyethnic states’ where immigrant groups remain distinct from the majority population. The right and freedom to continue a distinct way of life should allow them to integrate into the receiving country without giving up their traditions. Multiculturalism is based on the recognition of ethnic and cultural minorities and aims to enable these migrant communities to participate in society the same way as the majority population. While critical reception of multiculturalism has grown over the last 20 years, a potential ‘return of assimilation’ (Brubaker, 2001) and the ‘retreat of multiculturalism’ (Joppke, 2004) have been discussed both by scholars and policy-makers.

Literature on immigrant integration has mainly focused on assimilation and multiculturalism as two opposing modes of integration (Castles et al., 2014). These two concepts are both commonly used to describe the nature of integration as well as a certain type of integration policy based on particular modes of integration. In the commonly applied typology of Koopmans et al., (2005), assimilation and multiculturalism constitute opposite policy types based on how individual and group rights are assigned to immigrants.^3^ The dimension of individual rights captures all measures that guarantee immigrants’ equal access to civic rights, including access to citizenship. The second dimension of cultural group rights captures all measures that provide ethnic minorities with rights to preserve their culture and to foster plurality within society. Policies of assimilation are based on a restrictive assignment of rights to immigrants, and the idea of a one-sided adaption of immigrants to the receiving country. This type of integration policy demands immigrants to acculturate to the society of the receiving country in order to become more similar to natives in social and cultural terms. Immigrants are provided with only few rights on both dimensions, and citizenship is granted by the principle of descent. Cultural rights of minorities are limited in order to promote cultural monism in society. The access to rights and legal membership in the receiving country is highly conditional on predefined integration requirements. Policies of multiculturalism, on the opposing end of the dichotomous typology, are based on an extensive assignment of rights to immigrants, and the idea of preserving cultural diversity within the country of residence. This type is aimed at the support of cultural plurality in society, and provides immigrants with extensive rights, not only in the dimension of individual equality, but also in the dimension of cultural diversity. Citizenship is granted by the principle of birth right and with only few restrictions.

Much has been done both at political theory (e.g. Alba & Nee, 1997, 2003; Kymlicka, 1995, 2001) as well as empirical measurement (e.g. Brubaker, 2001; Favell, 2001; Joppke & Morawska, 2003; Koopmans et al., 2005) to conceptualize assimilation and multiculturalism as concepts of integration and specific integration policy regimes. Others aimed to find more universal and non-normative concepts of integration such as multilevel approach (Scholten & Penninx, 2016), different dimensions of integration (Heckmann et al., 2001), and integration as an on-going process (Penninx & Martiniello, 2004). With the rise of more quantitative comparative studies on integration policies, a series of measurements have been proposed to capture their empirical characteristics on different policy dimensions (see Goodman, 2015; Helbling, 2016). Indices allow the linkage of different policy types with their respective integration outcomes, and a comparison over countries and time. Nevertheless, fundamental research questions such as what type of policy regime produces more favourable integration outcomes remain empirically disputed, and results are inconclusive. While over recent decades both policy-makers and scholars were highlighting the importance of multicultural measures to foster immigrant integration, a substantial backlash on multiculturalism has occurred in the early 21st century (Brubaker, 2001; Entzinger, 2003; Joppke, 2004). Multicultural approaches were criticised for their failure to integrate immigrants into host societies. Main criticism focuses on the argument that multiculturalism is encouraging self-segregation, and therefore impedes integration into mainstream society (see Ersanilli & Koopmans, 2010; Fleischmann & Phalet, 2010; Koopmans, 2016). Meanwhile, other scholars suggest the contrary point of view, in underlining that countries with multicultural policies have performed better on several dimensions of immigrant integration than countries without such policies (see Bloemraad, 2006; Berry, Phinney, Sam, & Vedder, 2006; Wright & Bloemraad, 2012). Hence, empirical findings on the effect of integration policies on integration outcomes are not only mixed (see Koopmans, 2013) but also contradict one another diametrically (Manatschal, 2013).

These inconclusive findings reflect the challenge of developing sound research designs and empirical interpretations when faced with normative assumptions and mutually exclusive concepts of integration. While immigrant integration is commonly seen as a complex and multi-dimensional process, the development of non-normative conceptions remains challenging (Ager & Strang, 2008; Garcés-Mascareñas & Penninx, 2015). A limited concept validity of existing policy measurements makes it difficult to test outcomes of different integration policy regimes against each other and to draw reliable inference (Goodman, 2015). For that reason, the approach to find ideal types of different policy regimes has been criticised as too static and too normative (Helbling & Vink, 2013, p. 552). However, assimilation and multiculturalism have continued to be used and also revived under different terms (Finotelli & Michalowski, 2012, p. 235). This shows how difficult it is to construct meaningful theoretical comparisons without referring to some sort of typology. The tendency to focus empirical research upon narrow determinants and outcomes can offer limited remedy to the normativity trap. Integration policies are necessarily normative in nature by what they define as the policy problem and by how the remedy should look like. Therefore, social scientists need to “describe and explain these predominant and oppositional normative institutional and policy models, their actual impact on policies, and their effects” (Bader, 2007, p. 879). One way to do this, is to take the (normative) typology and link it with an analytical concept of causal mechanism that is assumed by a particular policy type. The essential aim of this paper is therefore to distinguish between normative expectations of immigrant integration and policy intervention as analytical concept. In order to develop useful causal explanations, the theoretical assertion about the causal effect of a policy output as independent variable must be paired with the specification of a causal mechanism explaining through which process integration outcomes are produced (see George & Bennett, 2005; Pawson & Tilley, 1997). However, the fundamental question of *how* specific policy types influence the policy target group and change its behaviour has rarely been addressed in comparative integration studies (Goodman, 2015). This neglect is all the more surprising since normative notions of integration are themselves behavioural expectations that refer to the process dimension of integration, but are rarely stated explicitly as a characteristic of a policy regime. Therefore, developing descriptive concepts of policy outputs is not sufficient to avoid a normative trap, and they need to be coupled with ideas on causal mechanisms found in different policy regimes such as assimilation and multiculturalism. These two concepts represent certain policy frames with a particular reconstruction of the policy problem and the subsequent remedy to it (Garcés-Mascareñas & Penninx, 2015). All policy interventions are based on a vision of how they achieve their outcomes. Hence, the extension of existing descriptive typologies into a mechanistic typology may help to bridge normative contents of integration (policy) concepts with the corresponding policy design and provides a coherent framework for empirical comparisons to evaluate the effectiveness of different integration policy regimes.

## Capability-aspiration framework to explain integration policy outcomes

The framework presented in Table 1 draws heavily upon prior theoretical reflections on migration and immigrant integration, but offers a new and comprehensive approach to immigrant integration based on actor-centered institutionalism (see Scharpf 1997). First, integration policies are conceptualized as institutions providing *incentives* and *opportunities* to immigrants. Second, the agency of immigrants is conceptualized as *aspirations* and *capabilities*. Finally, the overall framework presents integration policy outcomes as the intersection of intentional actors (immigrants) and regulatory institutions (policies).

**Table 1:** Conceptual comparison of intervention logics in integration policies

	Logic of conditioning	Logic of enabling
Policy Type	Assimilation	Multiculturalism
Concept of integration	Adaptation	Inclusion
Policy Frame		
Policy Problem	Under-aspiration	Under-capability
Causal Pathway	Aspirations lead to capabilities	Capabilities lead to aspirations
Policy Solution	Providing incentives to increase aspirations	Providing opportunities to increase capabilities

Public policies, such as immigrant integration policies, are specific institutions affecting the life of individuals much more directly than the formal design of the state that is commonly defined as institutions (see Pierson, 2006). Following the basic assumption of institutionalist theory, institutions are assumed to influence the preferences and strategies of individuals by stimulating or limiting behavioural options (see e.g. Hall & Taylor, 1996). Integration policies define rights and responsibilities for immigrants that are associated with their admission to the country.^4^ Particular policies consist of a set of rules that is assumed to facilitate integration by guiding immigrants’ behaviour. Although existing studies highlight the explanatory power of institutionalist theories for integration policies (see Boswell, 2008; Favell, 2001; Hansen, 2000, 2002; Koenig, 2007), the focus of the literature is predominantly on explaining path dependency and institutional change of these policies, and rarely focuses on the integration process as such. Furthermore, the application of institutionalist theories to the effect integration policies have on integration outcomes is largely divided into two different perspectives based on either the notion of incentives or opportunities.

One strand of literature applies the assumption that integration policies provide a particular incentive structure influencing immigrants’ preference formation that shapes their individual behaviour (Freeman, 2004; Hansen, 2012; Koopmans et al., 2005; Koopmans, 2010; Manatschal, 2013). A prominent application of this perspective is found in the question whether multicultural policies in combination with generous welfare states generate strong incentives to rely on social benefits instead of taking costly integration efforts (Hansen, 2012; Koopmans, 2010). Freeman (2004) provides a wholesale argument that integration policies provide an incentive structure in the main institutional domains of state, market, welfare and culture. The assumption is that the stronger policies incentivize integration, the more likely it becomes that immigrants will integrate.

Another strand of literature applies the assumption that integration policies provide a particular opportunity structure that either enables (or prevents) immigrants to participate in the receiving country (Bloemraad & Schönwälder, 2013; Cinalli & Giugni, 2011, 2013; Ireland, 2006; Kolbe, 2016; Koopmans & Statham, 2000; Koopmans, 2004). Opportunity structures can be defined as “institutions that define access and form of channels of participation for immigrants in mainstream society” (Kolbe, 2016, p. 421). The assumption is that the more integration opportunities are provided, the more likely it becomes that immigrants will integrate.

Nevertheless, incentives and opportunities are often used interchangeably and are not conceptualized as essentially different ideas. Since they resonate with different normative assumptions, the conceptual distinction of incentives and opportunities is central to disentangle normative from analytical concepts and to isolate causal mechanisms. Incentive-based approaches conceptualize policies as institutions altering the costs and benefits of different behavioural strategies. An incentive represents a motivation to perform a certain action and is therefore designed to alter preferences of what someone aims to do. Opportunity-based approaches conceptualize policies as institutions altering the available behavioural options. An opportunity represents a chance to achieve a certain goal and is therefore designed as a set of rules that limits or empowers actors. While incentives determine how attractive an action is, opportunities determine how feasible an action is.

While the incentive structure approach has found its main application in the social and economic integration of immigrants, the opportunity structure approach exists as a theory within the literature on social movements, and has been mainly applied to theorize the political integration of immigrants. These two strands of literature are both applying institutionalist ideas, but remain largely separate approaches and tackle different dimensions of the integration process. But although the two strands of literature tend to apply separate terminology, they often do not explicitely distinguish incentives and opportunities from each other. The comparison of these two perspectives is essential for the new capability-aspiration framework that takes the perspective that policies influence preferences with their incentives and simultaneously restrict or enhance the availability of opportunities.

In the next step, immigrants as the target group of integration policy are conceptionalized as intentional actors interacting with policies as institutions providing incentives and opportunities. Scholars have broadly acknowledged the nature of the integration process as a complex interaction between society and individual and integration outcomes cannot be studied solely by focusing on immigrants themselves or natives and characteristics of their institutions (Heckmann, 2006). An institutionalist perspective runs the risk of ignoring immigrants’ agency. A coherent framework for the analysis of integration outcomes requires a combination of both, individual intentional behavior and structural boundaries. As Hansen (2012, p. 8) noted previously, states must ensure “real opportunities and incentives to immigrants” and “immigrants themselves will have to want these opportunities”. In a similar vein, Freeman (2004) described immigrant integration as the intersection between immigrants’ strategies and the regulatory frameworks of the receiving state. Bridging the perspective of immigrants as intentional actors and the perspective of policies as institutions should therefore be combined to analyse outcomes of integration policies. Such a perspective is offered by actor-centered institutionalism that explains policy outcomes as the product of intentional actions of individuals and the institutional context structuring these actions (Scharpf, 2000). Moreover, individuals are characterised by specific capabilities and subjective action orientations (Scharpf, 1997). By capabilities, Scharpf refers to the power of actors to enact their decisions, and contains all resources that enable an actor to influence an outcome. Action orientations are the characteristic perceptions and preferences of an individual, in other words the aspirations to perform a particular action. Accordingly, integration capabilities and integration aspirations can be understood as subsets of more general capabilities and aspirations in life. The capability-aspiration framework essentially conceptualises immigrant integration outcomes as the product of immigrants’ *capabilities* and *aspirations* both shaped by integration policies.^5^

The first part of the framework consists of individual *capabilities* of immigrants to integrate into the society of the receiving country.^6^ Integration capabilities refer to individuals’ ability to realise integration aspirations given their constraints. Capabilities are the real opportunities to participate in society and to pursue one’s own goals, what someone is enabled *to do* and *to be*, whether or not someone chooses to do so. Accordingly, integration capability can be specified as the positive freedom to integrate, both the *doing* in the sense of the actual ability to participate in society, and the *being* in the sense of a personal identity as a full-fledged member of society. Together, these capabilities consist of all opportunities to acquire country-specific skills and resources, and the actual ability to convert them into participation and social belonging. To what extend someone is able to integrate depends strongly on individual resources and access to institutions. Immigrants’ capabilities based on factors such as discrimination or human capital have shown to be important for predicting integration outcomes (e.g. Rydgren, 2004; Van Tubergen, Maas, & Flap, 2004).

The second part of the framework consists of individual *aspirations* of immigrants to integrate into the society of the receiving country. Integration aspirations refer to individuals’ views of investing into integration as a desirable life project, and of undertaking efforts to acquire country-specific skills and resources to be able to fully take part in society – be it as a matter of social belonging, or as a mean to other personal ends. Following the actor-centered institutionalism of Mayntz and Scharpf (1995), aspirations consist of perceptions and interpretation of a situation (cognitive orientation), defining personal motivation, and individual identities. Based on previous integration literature, personal aspiration of immigrants is conceptualized as purposeful construction of the future (Boccagni, 2017). Therefore, integration aspirations consist of the perceptions and interpretation of the residence in the receiving country. These aspirations consist in a continuum ranging from no aspirations to invest into integration, to high aspirations to invest into integration. Individual aspirations are dependent on the subjective perception of institutional opportunity structures that defines how desirable it is for an immigrant to aim for participation and belonging in the receiving country. The concept of immigrants’ aspirations has been present in migration studies since decades and been mostly applied to different domains of integration such as education or employment (see Boccagni, 2017 for an overview). Immigrants’ aspirations have shown to be important for predicting socio-economic performance (e.g. Portes, McLeod, & Parker, 1978) and their general relevance have been recognized by integration studies (Freeman, 2004).

As outlined, in order to take part in society, someone needs to be able to integrate and needs to be willing to integrate – both capability and aspiration are therefore *necessary conditions* for individual integration. When immigrants arrive in the receiving country they have to make a place for themselves – to find a job, a new housing and establish a social network. Through the integration process they acquire competencies regarding key institutions of the receiving country that might function differently from the ones known in the country of origin.^7^ At their arrival, immigrants bring a set of resources and motivation with them to build up their new residence in the receiving country. Accordingly, integration outcomes constitute a function of the motivation to invest into a new life as a member of the receiving country (aspiration) on the one hand, and the opportunities that are available to invest beneficially on the other hand (capability). With a lack of aspiration, immigrants will not be sufficiently motivated to undertake costly integration efforts, even when there are opportunities available to do so. With a lack of capability, immigrants will fail to convert their motivation for integration into actual achievements, even if they strongly aspire to do so. Individual aspirations determine whether an immigrant chooses to exercise existing capabilities. Capabilities determine whether the aspirations are converted into actual achievements.

**Fig. 1:**
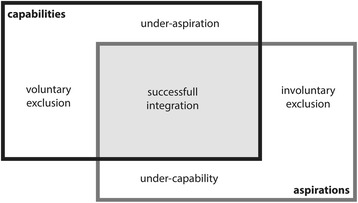
Capability-Aspiration-Framework for Integration Outcomes

Having laid out integration outcomes as the function of capabilities and aspirations, policies aiming to change the behaviour of immigrants necessarily affect at least one of these factors. Thus, the effect of integration policy outputs on corresponding policy outcomes is essentially mediated by their effect on individual capabilities and aspirations (see Fig. 1). Policy outputs^8^ (e.g. specific laws and measures) interact with its target group and larger society, and eventually produce a policy outcome (e.g. a change in the level of integration). Policies influence integration aspirations by altering the incentive structure, either by strengthening incentives to undertake integration efforts, or by weakening them and creating disincentives. Immigrant’s capabilities are influenced by integration policy which alters the opportunity structure, either by expanding opportunities and lowering barriers or by limiting opportunities and increase barriers.

Having established the policy focus of this contribution, it is more than likely that integration outcomes are susceptible of other explanations. Neither integration capabilities nor integration aspirations are solely policy-driven, but should be seen against the totality of different domains such as the state, market, welfare and culture of the receiving country (Freeman, 2004). The process of integration takes place under a given set of structural factors of the receiving country and individual factors of immigrants. Accordingly, both capabilities and aspirations are contingent on a range of individual factors, such as skills, and contextual factors, such as access to educational system and labour markets (see Van Tubergen et al., 2004). While a broad range of individual and contextual determinants of aspirations and capabilities exist that could be situated in an institutionalist framework, the remainder of this article will focus on how integration policy interventions are assumed to influence the complex interaction between capabilities and aspirations.

## Two logics of policy intervention: assimilation and multiculturalism

The new framework allows studying how specific policies influence immigrant integration outcomes and what logics of policy intervention they entail. This section seeks to establish assumed causal mechanisms of immigrant integration of assimilation and multiculturalism. Based on previous literature on immigrant integration the existence of two distinct principles of policy intervention woven into conceptual understandings of the integration process and policy designs is demonstrated.

Assimilation aims for social cohesion by the adaptation of immigrants to the characteristics of the native population, and multiculturalism aims for inclusion by recognition of cultural plurality. The two ideal types of integration policies can be characterised by how liberal or restrictive they are in the assignments of rights to immigrants (see Koopmans et al., 2005). The policy outputs of both types of integration policies are situated on the opposite sides of a continuum between a restrictive and an expansive assignment of rights to immigrants. Assimilation places a high bar in order to condition immigrants to become members of the society, whereas multiculturalism places a low bar in order to enable immigrants in their integration process. These different designs of policy outputs constitute the basis to localise different assumptions about the particular pathway of how policies influence the integration process of immigrants. Assimilation and multiculturalism not only have different normative aims and descriptive characteristics, but also imply particular ways of identifying how immigrants (should) integrate into the receiving country. By applying the framework to assimilation and multiculturalism, two distinct logics of policy intervention are derived.

**Fig. 2:**
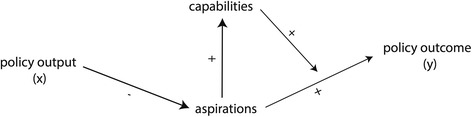
Logic of conditioning

In the perspective of assimilation, immigrant integration takes place as a process of adaptation to the receiving society. Immigrants are conceptualised as individuals with deficiencies (i.e. lacking country-specific skills) which makes it necessary to make costly efforts to ensure they become an integrated part of society. Integration is understood to be a burdensome process that needs efforts to make up for deficiencies of immigrants. The duty lies primarily with the immigrant that is expected to undertake integration efforts. Accordingly, policies of assimilation are designed to influence the interest of immigrants in adapting to the majority population. They are characterised by a restrictive provision of rights to immigrants. The provision of equal rights and citizenship is granted only after migrants successfully integrated into society. The theoretical expectation is that this restrictive and conditional provision of rights incentivizes immigrants to undertake integration efforts. The incentives of assimilation policies are expected to drive immigrants, who would otherwise stay within their ethnic groups, to acquire the necessary linguistic and cultural skills to integration. Higher aspirations in turn are expected to contribute positively to immigrants’ capabilities: If immigrants are interested enough in integration the opportunities will follow (see Fig. 2). Thus, any systematic underperformance of immigrants is expected to be the result of their limited aspirations to undertake the necessary integration investments. The essential policy problem that is to be addressed by policies of assimilation is the lack of aspirations of immigrants to undertake costly integration efforts. The role of rights is therefore the enhancement of aspirations, and rights function as a reward provided for integration achievements. With an extensive provision of rights, on the other hand, immigrants would lose incentives to undertake integration efforts, since benefits associated with rights don’t need to be earned anymore. The logic of policy intervention is a conditioning one, where integration is imposed on immigrants as a requirement for the granting of full rights. Policy intervention is aimed at placing sufficient incentives to increase aspirations of immigrants.

As illustrative example for this approach, the influential empirical study of Koopmans (2010)^9^ where the author argues that multicultural integration policies lead to social and economic marginalisation and therefore hamper the integration process. The assumed causal mechanism is based on the idea that multicultural policies “do not provide strong incentives for host-country language acquisition and for interethnic contacts” (Koopmans, 2010, p. 3) and “stimulate migrants to orient themselves towards their ethnic community, may have the unintended consequence of sustaining linguistic deficiencies and a lack of cultural ‘soft skills’.” (Koopmans, 2010, p. 10). Immigrants are assumed to lack country-specific skills that need to be acquired through the integration process. Policies are conceptualized as incentive structure. Immigrants are assumed to not invest into the skill acquisition in the absence of incentives. The study by Koopmans applies an institutionalist perspective of policies as *incentive*-structure influencing the *aspirations* of immigrants, here, whether to aim for the acquisition of country-specific skills and inter-ethnic contacts, or a preference for staying within ethnic communities.

**Fig. 3:**
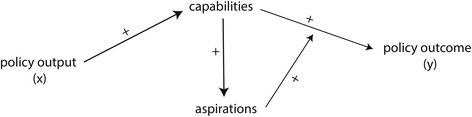
Logic of enabling

In the second perspective of multiculturalism, immigrant integration takes place as a process of inclusion. The acquisition of country-specific skills is meant to enable immigrants to participate as equals and to unfold their potential in the receiving society. Immigrants are perceived as individuals with potentials and an intrinsic motivation to take part in the society of the receiving country. The duty lies primarily with the receiving society that is supposed to provide opportunities for the participation of immigrants on equal terms. Accordingly, policies of multiculturalism are designed to influence the ability of immigrants to integrate. They are characterised by an extensive range of rights granted to immigrants. The theoretical expectation is that a liberal provision of rights to immigrants facilitates their participation. Rights provide resources and access to institutions that increase the ability of immigrants to realise equal participation. Thus, any systematic underperformance of immigrants is expected to be the result of their limited capabilities. More capabilities are expected in turn to contribute positively to immigrants’ aspirations: If immigrants are able to participate as equals, their perception of opportunities is given a boost and instil immigrants a belief that they can thrive in the receiving society (see Fig. 2).^10^ If rights are restricted, on the other hand, this will deprive immigrants of resources, and create barriers leading to exclusion and reduced capabilities. The essential policy problem that is to be addressed by policies of multiculturalism is therefore the lack of capabilites of immigrants to integrate into the receiving society. The role of rights is primarily the enhancement of capabilities, and rights function as a catalyst for integration efforts. The logic of policy intervention in multiculturalism is an enabling one, where integration is a voluntary act that is facilitated by the provision of resources and facilitated access to institutions. This logic is further expressed by the recognition of cultural variety as a central aim of multiculturalism that should provide the necessary psychological and symbolic resources for a more even playing field. Policies of multiculturalism are therefore assumed to function as a catalyst to achieve integration by enabling a positive dynamic between individual migrants and the receiving society (Fig. 3).

As illustrative example for this approach is the prominent research of Irene Bloemraad on the political integration of immigrants (Bloemraad, 2006). The starting point is that immigrant integration outcomes are “fundamentally about the reception we give them” (Bloemraad, 2006, p. 2). In the theoretical framework government policies are characterized as opportunity structures that provide “material and symbolic resources that assist in political incorporation” (Bloemraad, 2006, p. 9). The assumed causal mechanism that follows is that these resources influence the “mobilization capacity” of immigrant communities (Bloemraad, 2006, p. 106). This study applies an institutionalist perspective of policies as *opportunity*-structure influencing the *capabilities* of immigrants, whether immigrants are able to political mobilization and participation. Whether immigrants decide to participate is seen depending primarily on the resources available to them.

Multiculturalism and assimilation both operate, at least implicitly, with ideas of integration capabilities and integration aspirations, but they differ in regard to the assumed underlying causal mechanism. Assimilation policies are aimed at an assumed lack of aspirations, and multicultural policies are aimed at an assumed lack of capabilities. Each approach measures variation in the same causal space, but is based on different theoretical representations of this causal space. Without revealing the two logics of policy intervention, there results a lack of comparability of research findings, and this hinders a thorough empirical comparison of both approaches. The inclusion of aspiration and capabilities to the study of immigrant integration provides a coherent framework to systematise assumptions about the integration process, and reveals two rationales of integration policy intervention.

After the presentation of the capability-aspiration framework, the scope and benefits of its application are discussed. The openness and generality of the framework allows for a potentially broad scope of application. First and foremost, the logics of policy intervention refer to receiving countries that enact systematic integration policies based on a particular mode of integration that can be classified as assimilation or multiculturalism. The framework allows covering all dimensions of integration and integration policy rendering it applicable to a broad range of outcomes such as political participation (legal-political) or employment (socio-economic) or social belonging (cultural-religious).^11^ All dimensions of the integration process can be conceptualized by assumptions of actor-centered institutionalism with policies creating particular incentive and opportunity structures. Integration policy takes place on different state levels from local to supranational polities. Since immigrants are influenced by all levels of policy-making, all policies provide institutional settings described by the proposed framework.

The essential feature of actor-centered institutionalism is that it allows for the combination agency and structure within a uniform explanatory framework. Furthermore, the new framework allows learning how different ideas about the normative nature of the integration process result in particular types of policy intervention and what causal mechanism they imply. This might not only facilitate to disentangle contradicting findings on integration policy effectiveness but may also minimize the implicit transmission of outdated and stereotypical attributions of policy regimes on the interpretation of policy outcomes. The two concepts of assimilation and multiculturalism imply different specifications of the policy problem, and identify different causal pathways behind the relationship between the policy output and the policy outcome.

## Conclusions

The comparative analysis of immigrant integration policies face opposing normative notions of integration and a lack of concept validity when it comes to empirical measurements of policies. As a result, empirical evidence on which types of integration policy contribute under which circumstances to favorable integration outcomes remains inconclusive and disputed.

The argument lined out in this article is that the confusion in the literature is at least partially the result of the underspecification of causal mechanisms regarding different types of integration policies. How specific policies are assumed to intervene into the integration process remains often a blackbox. Therefore, these assumed mechanisms need to be specified in a systematic manner and the nature of integration process linked with a concept of policy intervention. By applying the perspective of actor-centered institutionalism to integration policy outcomes a new coherent framework distinguishing integration capabilities from integration aspirations is presented. In this perspective, integration can be conceptualised as a function of individual aspirations to become a full member of the receiving country, and the individual capability to translate these aspirations into effective integration. In order to participate in and feel belonging to the receiving country, immigrants require both the motivation to integrate and the ability to do so.

The application of this capability-aspiration framework to the policy design of multiculturalism and the policy design of assimilation illustrates how these two opposing policy types differ in their assumption about the policy problem, the policy solution, and the logic of policy intervention. Different policy regimes for immigrant integration address different aspects of the integration process and the dynamics between the individual immigrant and the society at large: assimilation seeks to strengthen the aspiration of immigrations, whereas multiculturalism seeks to strengthen the capabilities of immigrants. The integration policy problem is either seen as under-aspiration in the case of assimilation, or as under-capability in the case of multiculturalism. Accordingly, in the perspective of assimilation, integration outcomes can be improved by fostering immigrants’ aspirations. In the perspective of multiculturalism, integration outcomes can be improved by fostering immigrants’ capabilities. The fundamentals of the proposed framework are considering capabilities and aspirations as two sides of the integration coin, and the assignment of rights to immigrants as a potential double-edged sword. The study illustrates why assimilation and multiculturalism remain the main concepts of immigrant integration: they are based on two different visions of the integration process shaping two opposing premises about the policy problem and the remedy to it. Assimilation and multiculturalism form public philosophies of integration that structure and constrain their specific policy interventions. This article argues that assimilation is an incentive-based theory and multiculturalism is an opportunity-based theory.

Nevertheless, the chosen approach of actor-centered institutionalism may ignore or conceal certain aspects of integration processes. Other explanatory approaches focussing on factors such as ethnic discrimination or educational resources are complementary to the proposed framework. Considering integration outcomes as a product of capabilities and aspirations is illuminating for an institutionalist perspective on integration policy outcomes and allows identifying specific policy intervention logics as demonstrated in this article. The benefits of this study are threefold. Firstly, from a theoretical perspective, the capability-aspiration framework provides a conceptual tool for the analysis of the effectiveness of policy interventions by combining the idea of an agency-structure relationship of actor-centered institutionalism with the comparative study of immigrant integration. Instead of a full-fledged explanation model, the capability-aspiration framework is a research heuristic on a high level of abstraction that allows the identifying and arranging of different explanatory factors relevant for immigrant integration outcomes in different dimensions.

Secondly, from an empirical perspective, I illustrate how the contradicting findings in the literature can be disentangled, and elaborate on differences in the policy problem, the policy solution, and distinct causal mechanisms that have only been assumed implicitly in existing studies. While there are useful normative and descriptive typologies of integration policy regimes, the capability-aspiration framework offers a mechanistic typology of *how* policies are expected to influence integration outcomes of immigrants. The framework may help to translate normative notions of integration into useful analytical concepts of policy intervention to identify complex causal relationships.

Thirdly, from a practical perspective, the new framework can help to understand the motivation and reasoning of politicians regarding specific integration policy interventions. Gaining certainty about the (perception of the) policy problem will allow policy-makers to draft more targeted policy intervention by anticipating the effects on immigrants’ capabilities and aspirations. The framework allows establishing clear links between the notion of integration and the corresponding policy intervention. Therefore, the proposed heuristic may provide a useful tool, sharpening the understanding of integration policy effectiveness.

Once established that integration outcomes are a complex interaction of aspirations and capabilities and that integration policy effectiveness depends on a complex interaction of incentives and opportunities, we can start building up systematic knowledge of how they interact. If this conceptual distinction is ignored or incentives and opportunities are taken as the same thing, we risk to reproduce the normativity trap and to miss the complex interaction of aspirations and capabilities. Future research should further specify and investigate the causal mechanisms outlined in this study in order to solve the puzzle of contradicting findings in existing literature on immigrant integration. Empirical studies on policy effectiveness may gain explanatory power if they specify capabilities and aspirations as mediating factors. The major challenge for future research will be to find empirical strategies to measure capabilities and aspirations in specific dimensions of immigrant integration in order to test the different logics of policy intervention against each other. The capability-aspiration framework provides a research heuristic meant to facilitate comparative studies over mutually exclusive approaches to immigrant integration, and proposes to focus on assumed but broadly untested causal relationships in different types of integration policy regimes.

## Footnotes

1 The terms assimilation, acculturalisation, incorporation, pluralism or multiculturalism are commonly used interchangeably with the notion of integration. These notions are often vaguely specified and overlapping in their definitions. They are applied as synonyms but also as mutually exclusive concepts. The notion applied in this article refers to integration in a broad sense as the part of the migration process following arrival in the receiving country (Hammar, 1985).

2 Multiculturalism is often used to describe the increasing ethnic diversity in a country as a result of immigration. Therefore, it is important to distinguish this conception from the one used here as a normative concept of integration and a subsequent model of policy.

3 Integration can be conceived of as both an individual as well as group phenomenon. The theoretical argument of this article should speak to both concepts of integration. I use the term immigrant integration to refer to both types of integration processes.

4 Admission policies as rules about entry and stay of immigrants are often referred to ‘immigration policy’, whereas integration policies are referred to as ‘immigrant policy’ (see Hammar, 1985).

5 For the application of capabilities and aspirations to migration theory see Carling (2002) and De Haas (2009, 2011).

6 The so-called ‘capability approach’ originated in the work of Amartya Sen (1999), who defined human capability as the ability of human beings to lead lives they have reason to value, and to enhance the substantive choices they have.

7 Integration as a process is meant to conceptualize it as an on-going development instead of a result once achieved (Penninx & Martiniello, 2004). The conceptualization of immigrant integration as a social process that is dynamic and multidimensional in nature has become broad consensus in integration studies (see e.g. Heckmann, Köhler, Peucker, & Reiter, 2010).

8 The effect of formal outputs on outcomes often depends on how a law or measure is implemented. For the theoretical framework is assumed for simplisticity reasons that policy outputs are also implemented.

9 This study is among the most cited empirical studies on integration policy outcomes (127 citations on the Social Science Citation Index and 390 on Google Scholar).

10 See also the ‚capacity to aspire’ by Appadurai (2004).

11 These three dimensions of integration processes have been extensively described by Penninx and Garcés-Mascareñas (2016).
